# Sugarcane Polyphenols Improve Depressive-like Behavior in CUMS Mice by Promoting the MAPK/ERK Signaling Pathway and Inhibiting NLRP3 Inflammasome Pyroptosis

**DOI:** 10.3390/foods15132322

**Published:** 2026-06-30

**Authors:** Xue Wang, Jiapeng Song, Zhongmei He, Jianming Li, Yan Zhao, Ying Zong, Jianan Geng, Jia Zhou, Junkoo Yi, Weijia Chen, Rui Du

**Affiliations:** 1 College of Chinese Medicinal Materials, Jilin Agricultural University, Changchun 130118, China; 13889862812@163.com (X.W.); 15637212726@163.com (J.S.); heather78@126.com (Z.H.); m15568781138@163.com (J.L.); zhaoyan@jlau.edu.cn (Y.Z.); zongying7699@126.com (Y.Z.); gengjianan@jlau.edu.cn (J.G.); 007152@jlau.edu.cn (J.Z.); 2 Jilin Provincial Engineering Research Center for Efficient Breeding and Product Development of Sika Deer, Changchun 130118, China; 3 Key Laboratory of Animal Production and Product Quality and Security, Ministry of National Education, Changchun 130118, China; 4 Division of Animal Science, School of Animal Life Convergence Sciences, Anseong City 12646, Republic of Korea; junkoo@hknu.ac.kr

**Keywords:** sugarcane polyphenols (SP), depression, MAPK, NLRP3 inflammasome, pyroptosis

## Abstract

Sugarcane polyphenols (SP) are investigated for their antidepressant potential using a CUMS-induced mouse model and a corticosterone-induced neuronal injury cell model. Results demonstrate that SP alleviates depressive-like behaviors, inhibits hippocampal neuronal apoptosis, and reduces neuroinflammation. Mechanistically, SP activates the MAPK/ERK pathway, which in turn suppresses NLRP3 inflammasome-mediated pyroptosis; this effect is attenuated by the MAPK/ERK inhibitor PD98059. Furthermore, SP synergizes with the caspase-1 inhibitor VX-765 to inhibit pyroptosis.

## 1. Introduction

Depression is a prevalent mental disorder characterized clinically by anhedonia, low energy, diminished self-esteem, impaired concentration, and recurrent suicidal ideation [1]. According to the World Health Organization, over 350 million people worldwide are affected by depression [2]. It ranks as the second leading cause of global disease burden and is projected to become the primary contributor to disability by 2030 [3]. However, the precise mechanisms underlying depression and the action of antidepressants remain unclear. Most current antidepressants target monoamine neurotransmitter systems, including selective serotonin reuptake inhibitors [4]. Different types of antidepressants include selective serotonin reuptake inhibitors [5], serotonin and norepinephrine reuptake inhibitors [6], norepinephrine and specific serotonin antidepressants, tricyclic antidepressants, and monoamine oxidase inhibitors [7]. These drugs are often associated with side effects such as anxiety, gastrointestinal disturbances, decreased alertness, and sexual dysfunction [8]. Therefore, it is important to develop new antidepressants that are affordable, have few side effects, are effective, and are safe.

Sugarcane (*Saccharum sinensis* Roxb.) is a perennial herbaceous plant belonging to the Poaceae family [9]. In modern times, sugarcane is rich in various active ingredients such as polyphenols, amino acids, and vitamins, and has been proven to have good medicinal value [10,11]. Sugarcane polyphenols have excellent antioxidant [12], blood sugar-lowering, lipid-lowering, antibacterial, and antitumor effects [13], and are effective in treating a range of diseases caused by oxidative stress damage, such as aging, diabetes, inflammation, and tumors [14]. Sugarcane polyphenol was reviewed and approved by the National Health Commission on 23 May 2022, and officially included in the new food ingredient catalog. Its safety assessment strictly followed the Food Safety Law and the Measures for the Safety Review of New Food Ingredients. The United States has classified sugarcane polyphenols as “generally recognized as safe” (GRAS) substances [15], the European Union allows their use as food ingredients, Australia and New Zealand manage them as ordinary foods, and Taiwan has already used them as food ingredients. However, research on the antidepressant activity and mechanism of action of sugarcane polyphenols is limited.

Neural injury often triggers neuroinflammation, a key contributor to the pathogenesis of various neurological disorders. Excessive inflammation can induce abnormal morphological changes and neuronal damage, thereby promoting depressive-like behaviors. The Mitogen-Activated Protein Kinase (MAPK) family, which includes ERK1/2, JNK, and p38, plays important roles in phosphorylating serine and tyrosine residues in response to diverse stimuli [16]. While ERK1/2 is primarily activated by growth factors, JNK and p38 are more responsive to oxidative stress and cytokines [17]. These pathways can lead to activation of transcription factors such as NF-κB and are involved in inflammation and stress responses [18].

Overexpression of the MAPK/ERK pathway in the hippocampus may reduce synaptic plasticity and impair memory and learning [19]. Chronic stress has been shown to activate the p38/JNK inflammatory pathway while suppressing the neurotrophic ERK pathway, contributing to neuronal injury [20]. Inhibiting MAPK/ERK signaling may protect hippocampal neurons, reduce apoptosis, and alleviate depressive symptoms. Moreover, the MAPK pathway influences synaptic plasticity and the expression of hippocampal plasticity markers, as observed in mice with depression-like behavior following prolonged morphine withdrawal [21,22].

Polyphenols are known to modulate the MAPK signaling pathway at multiple levels [23]. Through regulation of MAPK, polyphenols can induce apoptotic responses and exert anti-inflammatory and anticancer effects [24]. Specific polyphenols such as catechins and quercetin activate MAPK, p38, ERK1/2, and JNK in a dose-dependent manner, thereby inhibiting genes like the plasminogen activator inhibitor-1 (PAI-1) [25].

Previous studies have established that the NLRP3 inflammasome complex is intimately associated with inflammatory processes. Its activation triggers the release of inflammatory mediators and propagates the downstream inflammatory cascade [26]. Inflammation in depression is also closely linked to the NLRP3 inflammasome complex. Its activation triggers ASC recruitment, caspase-1 activation, and the maturation and release of IL-1β, alongside cleavage of GSDMD to GSDMD-N, ultimately promoting depressive-like behavior [27,28].

Ameliorating pyroptosis has shown anti-inflammatory and neuroprotective effects, alleviating depression-like symptoms [29]. This includes reducing NLRP3 expression and enhancing hippocampal neurogenesis markers such as DCX [30].

Polyphenols can downregulate the NLRP3 inflammasome pathway at multiple signaling steps. Beyond antioxidant activity, they regulate NLRP3 activation, supporting their potential in preventing and managing inflammation-driven metabolic diseases [31].

Since sugarcane polyphenols have a good therapeutic effect on inflammation and nerve damage caused by oxidative stress damage, we hypothesize that sugarcane polyphenols may be a multi-target MAPK activation inhibitor. Therefore, this study evaluated the antidepressant activity of sugarcane polyphenols by establishing an in vitro CORT model and an in vivo CUMS model. By adding MAPK/ERK inhibitors (PD98059) and pyroptosis inhibitors (VX-765), we delved deeper into the mechanism of action of sugarcane polyphenols in treating depression. This provides new directions and insights into how sugarcane polyphenols can be used as a new food resource to treat depression.

## 2. Materials and Methods

### 2.1. Chemicals and Reagents

Sugarcane: Guangxi black-skinned sugarcane. Fluoxetine hydrochloride and corticosterone (Source: Yeyue, Shanghai, China); PD98059 (Source: Yeyue, Shanghai, China). DMEM medium (Wanleibio, Shenyang, China); phosphate-buffered saline (PBS) (Wanleibio, Shenyang, China); high-quality fetal bovine serum (FBS) (Wanleibio, Shenyang, China). TBST solution (Wanleibio, Shenyang, China), transmembrane buffer (Wanleibio, Shenyang, China). Trypsin solution (EDTA-free) (Wanleibio, Shenyang, China), skim milk powder (Wanleibio, Shenyang, China), Color Pre-stained Protein Marker VII (HUABIO, Hangzhou, China). Anti-NLRP3 rabbit (HUABIO, Hangzhou, China), bovine serum albumin (BSA) (Wanleibio, Shenyang, China), anti-ASC rabbit (HUABIO, Hangzhou, China), anti-Caspase-1 rabbit (HUABIO, Hangzhou, China), anti-IL-1β rabbit (HUABIO, Hangzhou, China), anti-P38 rabbit (HUABIO, Hangzhou, China), anti-P-P38 rabbit (HUABIO, Hangzhou, China), anti-ERK rabbit (HUABIO, Hangzhou, China), Tris-glycine SDS-PAGE electrophoresis buffer (Wanleibio, Shenyang, China),anti-P-ERK rabbit (HUABIO, Hangzhou, China), anti-JNK rabbit (HUABIO, Hangzhou, China), and anti-P-JNK rabbit (HUABIO, Hangzhou, China), anti-GAPDH rabbit (HUABIO, Hangzhou, China), Polyvinylidene fluoride (PVDF) membrane (MerckMillipore, Darmstadt, Germany),anti-GSDMD-N rabbit (HUABIO, Hangzhou, China), HRP-labeled goat anti-rabbit IgG (HUABIO, Hangzhou, China), penicillin-streptomycin solution, 4% paraformaldehyde (PFA) (Wanleibio, Shenyang, China), Alexa Fluor 488-labeled goat anti-rabbit IgG (HUABIO, Hangzhou, China), and DAPI staining reagent (HUABIO, Hangzhou, China).

### 2.2. Preparation of Polyphenols from Sugarcane

As far as we know, extraction parameters (including solvent concentration, feed-to-solvent ratio, ultrasonication time, and ultrasonication temperature) are crucial because they influence the extraction rate of polyphenols. Since polyphenolic compounds contain hydroxyl groups in their structure and exhibit a certain degree of polarity, ethanol, which has good polarity and excellent solubility for phenolic compounds, is chosen as the extraction solvent for sugarcane polyphenols due to its non-toxic nature and low cost. The use of X-5 macroporous resin for the purification of sugarcane peel polyphenols can improve the purity of polyphenols and is feasible. Therefore, a single-factor experiment was conducted using X-5 macroporous resin for purification. Purification parameters (including loading concentration, volume fraction, flow rate, and volume) are important because they affect the purity of polyphenols. Use Folin phenol reagent to test the purity of sugarcane polyphenols with a microplate reader [32]. Based on single-factor experiments, a response surface analysis experiment with four factors and three levels was designed according to the principles of central composite design.

### 2.3. Extraction and Content Determination of Sugarcane Polyphenols

Sugarcane was dried at 40 °C, ground, and sieved. The resulting powder was stored at low temperature for later use. A precise amount of sugarcane powder was weighed, mixed with a certain concentration of ethanol solution at a designated solid-to-liquid ratio, and extracted by ultrasonication. After cooling, the mixture was filtered under vacuum. The filtrate was diluted to 50 mL, allowed to stand for 20 min, and then 0.02 mL of the test solution was accurately pipetted into a 10 mL volumetric flask. The absorbance was measured according to the method described above, and the polyphenol content was calculated using the regression equation. The polyphenol extraction yield was calculated according to formula:

Polyphenol yield (%) = (Y + 0.0231) × 25000 × 10^−6^ × 100%/(0.1401 × m)

where Y is the absorbance and m is the mass (g) of sugarcane powder.

### 2.4. Single-Factor Optimization and Response Surface Analysis of the Extraction Process

In the optimization of the extraction process, single-factor experiments were first conducted to investigate the effects of ethanol concentration (30%, 40%, 50%, 60%, 70%), solid-to-liquid ratio (1:10, 1:15, 1:20, 1:25, 1:30 g/mL), extraction temperature (40, 50, 60, 70, 80 °C), and ultrasonic time (5, 10, 20, 30, 40 min) on the yield of polyphenols from sugarcane peel. While keeping other conditions constant, only one variable was changed at a time. After ultrasonic extraction, the mixture was filtered under vacuum, diluted to volume, and the polyphenol content was determined. Based on the results of the single-factor tests, key factors and their levels were selected for optimization using the Box–Behnken Design (BBD) response surface methodology. Design Expert 7.0 software was employed to develop an experimental plan with four factors and three levels, comprising 29 runs including five center points to estimate experimental error. The run order was randomized to minimize systematic bias. With ethanol concentration (A), solid-to-liquid ratio (B), extraction temperature (C), and ultrasonic time (D) as independent variables, and polyphenol yield (Y) as the response, a mathematical model was established. Regression analysis was performed, response surface plots were generated, and the optimal extraction parameters were determined.

### 2.5. Single-Factor Optimization and Response Surface Analysis of the Purification Process

In the purification process optimization, the effects of sample loading concentration (2, 4, 6, 8, 10 mg/mL), ethanol elution concentration (50%, 60%, 70%, 80%, 90%), eluent flow rate (1, 2, 3, 4, 5 BV/h), and eluent volume (1, 2, 3, 4, 5 BV) on polyphenol purity were examined sequentially. A static adsorption method was applied to determine the optimal level for each factor. Based on the single-factor results, four factors—sample loading concentration, ethanol concentration, flow rate, and eluent volume—were selected for further optimization using the BBD response surface methodology. A four-factor, three-level experimental matrix of 29 runs, including five center points, was designed with Design Expert 7.0 software, and the runs were executed in random order. With sample loading concentration (A), ethanol elution concentration (B), eluent flow rate (C), and eluent volume (D) as independent variables, and polyphenol purity (Y) as the response, regression analysis was conducted and response surface plots were generated to determine the optimal purification conditions for macroporous resin.

### 2.6. Material Identification

Methanol, acetonitrile, and formic acid (HPLC grade) were obtained from Thermo Fisher Scientific (Beijing, China). Analysis was performed on a Thermo Vanquish UPLC (Germering, Germany) coupled to a Q Exactive HFX mass spectrometer (Bremen, Germany).

For extraction, frozen samples (50–100 mg) were thawed at 4 °C, mixed with 1 mL of water/acetonitrile/isopropanol (1:1:1, *v*/*v*/*v*), vortexed for 60 s, and ultrasonicated at low temperature for 30 min. After centrifugation at 12,000 rpm (4 °C, 10 min), the supernatant was held at −20 °C for 1 h to precipitate proteins and centrifuged again. The supernatant was vacuum-dried, reconstituted in 200 μL of 50% aqueous acetonitrile, vortexed, and centrifuged at 14,000 rpm (4 °C, 15 min). The clear supernatant was used for LC-MS analysis.

Chromatographic separation employed a Waters HSS T3 column (Taunton, MA, USA) (100 × 2.1 mm, 1.8 μm) at 40 °C. Mobile phase A was 0.1% formic acid in water, and B was 0.1% formic acid in acetonitrile, at 0.3 mL/min. Injection volume was 2 μL. Gradient: 0–1 min, 100% A; 1–12 min, linear to 5% A; 12–13 min, 5% A; 13–13.1 min, return to 100% A; 13.1–17 min, re-equilibration. Samples were kept at 4 °C in the autosampler and injected randomly. QC samples were interspersed to monitor system stability.

Mass spectrometry was performed in positive and negative ESI modes. Parameters: sheath gas 40 arb, auxiliary gas 10 arb, spray voltage ±3000/2800 V, ion transfer tube 320 °C, heater 350 °C. Full MS-ddMS^2^ acquisition covered *m*/*z* 70–1050 at 70,000 resolution (full MS) and 17,500 (MS/MS).

### 2.7. Cell Culture and Cell Concentration Screening

This experiment used the HT22 cell line provided by Wuhan Puno Sai Life Technology Co., Ltd. (Wuhan, China). The HT22 cell culture medium comprises: 10% heat-inactivated foetal bovine serum, 1% penicillin and streptomycin, and DMEM. HT22 cells were cultured in an incubator maintained at 37 °C with 5% CO_2_. Cell concentration screening was performed by adding solutions of varying SP concentrations to culture flasks. HT22 cells were divided into:

Control group
Model group: CORT group (100 μM) [33]
Low-dose group: CORT + SP (25 μg/mL)
High-dose group: CORT + SP (100 μg/mL)
Cell viability was ultimately assessed using the CCK-8 assay.

### 2.8. Intracellular ROS Detection

Logarithmic-phase HT22 cells were seeded into black 96-well plates (5 × 10^4^ cells/well). After attachment, cells were exposed to 100 μM corticosterone (CORT) for 12 h to establish oxidative stress injury. The medium was then replaced with fresh medium containing various concentrations of sugarcane polyphenols, and incubation continued for another 12 h. Subsequently, cells were loaded with 10 μM DCFH-DA in serum-free medium and incubated at 37 °C in the dark for 30 min. After three gentle washes with pre-warmed PBS, fluorescence images were captured using a fluorescence microscope.

### 2.9. PI Hoechst Staining

Logarithmic-phase HT22 cells were seeded into 6-well plates (1 × 10^5^ cells/well) and cultured until full adhesion before drug treatment. After treatment, the medium was removed, and cells were rinsed twice with pre-warmed PBS. Each well then received 5 μL of Hoechst 33342 (1 mg/mL), 5 μL of PI (1 mg/mL), and 1 mL of serum-free staining buffer. The plate was gently swirled, wrapped in aluminum foil, and incubated at 37 °C for 20 min. Cells were washed three times with ice-cold PBS to remove unbound dye, kept moist with a small volume of PBS, and immediately imaged using an inverted fluorescence microscope with appropriate filter sets.

### 2.10. Laboratory Animals and Experimental Design

Female C57BL/6 mice (6–8 weeks old) were obtained from Changchun Isis Laboratory Animal Technology Co. (Changchun, China). and housed in the Laboratory Animal Research Center of Jilin Agricultural University. Animals were kept in cages with free access to food and water under controlled conditions (20–22 °C, 50–60% humidity, 12 h light/dark cycle). All procedures were approved by the Ethics Committee of Jilin Agricultural University (Approval No. 20240827001) and conducted in accordance with the National Guidelines for the Care and Use of Laboratory Animals. Surgeries were performed under isoflurane anesthesia, and all efforts were made to minimize suffering.

### 2.11. Experiment 1: Effect of SP on CUMS-Induced Depressive Model Mice

After one week of acclimatization, C57BL/6J mice were randomly divided into five groups (*n* = 10): control group (Control), model group (CUMS), positive control group (CUMS + FLU, 20 mg/kg), low-dose SP group (SP, 0.25 g/kg), and high-dose SP group (SP, 1 g/kg). Except for the control group, the other four groups were subjected to an 8-week chronic unpredictable mild stress (CUMS) procedure. The CUMS paradigm included a series of unpredictable mild stressors such as 24 h food deprivation, 24 h water deprivation, forced swimming, 12 h light/dark cycle reversal, cage tilting, and restraint. SP and fluoxetine were administered via gavage starting from the 5th week of CUMS modeling.

### 2.12. Experiment 2: Effect of the MAPK Inhibitor PD98059 on the Action of SP

After one week of acclimatization, C57BL/6J mice were randomly divided into four groups (*n* = 10): control group (Control), model group (CUMS), treatment group (SP, 1 g/mL), and inhibitor group (SP, 1 g/mL + PD98059, 10 mg/kg). Starting from the 5th week of CUMS modeling, the treatment and inhibitor groups received daily gavage administration of SP for four weeks. Additionally, beginning from the 7th week, the inhibitor group received daily intraperitoneal injections of the ERK inhibitor PD98059 for two weeks. Subsequent behavioral tests, sample collection, and analysis procedures followed the same protocol as in Experiment 1.

### 2.13. Experiment 3: Effect of the Pyroptosis Inhibitor VX-765 on SP Activity

After one week of acclimatization, C57BL/6J mice were randomly divided into four groups (*n* = 10): control group (Control), model group (CUMS), treatment group (SP, 1 g/mL), and inhibitor group (SP, 1 g/mL + VX-765, 10 mg/kg). Starting from the 5th week of CUMS modeling, both the treatment and inhibitor groups received daily oral gavage of SP for four weeks. Additionally, beginning from the 7th week, the inhibitor group received daily intraperitoneal injections of the caspase-1 inhibitor VX-765 for two weeks. Subsequent behavioral tests, sample collection, and analysis steps followed the same procedure as in Experiment 1.

### 2.14. CUMS Modeling Schedule

Animal Experiment Protocol
Week 0: Adaptation Period.

Mice will be housed under standard conditions with free access to food and water for 1 week to allow acclimatization.

Chronic Unpredictable Mild Stress (CUMS) Modeling Phase (Weeks 1–8).

CUMS modeling will be conducted continuously for 8 weeks. Stress procedures will begin daily at 9:00 a.m. The following stressors are included in the paradigm: food deprivation for 12 h, ice-cold water swimming (4 °C) for 5 min, water deprivation for 12 h, warm water swimming (28 °C) for 10 min, cage shaking for 10 min, reversed light/dark cycle, damp bedding for 12 h, novel object exposure for 12 h, noise exposure for 12 h, tail suspension for 10 min, cage tilt (45°) for 12 h, and heat stress for 5 min. The specific schedule for mold making is shown in Table 1.

### 2.15. Sucrose Preference Test (SPT)

Before the experiment, mice were acclimated to 1% sucrose solution for 24 h. Following 24 h of food and water deprivation, mice were given access to two bottles containing either water or 1% sucrose solution for 2 h. Sucrose preference was calculated as: sucrose consumption/(sucrose consumption + water consumption) × 100% [34].

### 2.16. Tail Suspension Test (TST)

The tail suspension test (TST) was performed by suspending mice 50 cm above the floor for a total duration of 6 min. The first 2 min served as an acclimation period, after which immobility behavior was recorded and quantified during the subsequent 4 min using a video camera and automated scoring system [35].

### 2.17. Forced Swimming Test (FST)

Place the mouse in a transparent cylinder with a diameter of 10 cm and a height of 30 cm, filled with 20 cm of water. Using a camera and counter, record the total duration of immobility during the last 4 min of a 6 min activity period [36].

### 2.18. Open Field Test (OFT)

The open field test (OFT) was conducted in a black-walled arena (80 × 80 × 80 cm) under dim lighting. Mice were placed in the center and allowed to explore freely for 5 min. Locomotor activity, including immobility time and time spent in the central zone, was recorded and analyzed using a ceiling-mounted camera and automated tracking system [37].

### 2.19. Elevated Plus Maze (EPM)

The elevated plus maze (EPM) is commonly employed to evaluate antidepressant efficacy and validate depression models. The apparatus comprises two open and two closed arms. Each mouse was placed in the center of the maze and allowed to explore freely for 5 min. The number of entries into the open arms, as well as the time spent and distance traveled in the open arms, were recorded using an overhead camera. All tests were conducted in a quiet environment. Feces were removed and the apparatus was cleaned with alcohol between trials to eliminate olfactory cues [38].

### 2.20. Morris Water Maze (MWM)

The Morris water maze (MWM) consisted of a circular pool (diameter: 120 cm) filled with water (22 ± 2 °C) containing a hidden platform submerged 1–2 cm below the surface. Mice were trained for 5 consecutive days to locate the platform. Swimming trajectories and escape latency were monitored using a ceiling-mounted camera connected to the Smart 3.0 video tracking system (Panlab, Cornellà de Llobregat, Spain). The frequency of target quadrant crossings was also analyzed [39].

### 2.21. Radial Arm Maze (RAM)

The radial arm maze (RAM) comprised a central platform (30 cm diameter) surrounded by eight arms (70 × 10 cm, 10 cm high walls), enclosed in a soundproof and lightproof chamber. Mice were trained for 8 consecutive days to locate food pellets placed at the end of each arm. During each trial, all eight arms were baited, and mice were allowed to explore freely until all pellets were collected or 10 min had elapsed. Arm entries and movement trajectories were recorded using an overhead camera connected to the Smart 3.0 video tracking system (Panlab, Spain). The bait locations remained constant throughout the experiment [40].

### 2.22. Measurement of 5-HT, BDNF, CORT, IL-1β, and IL-10 Expression Levels in Mouse Serum and Hippocampal Tissues

After the final behavioral test, mice were fasted for 12 h with free access to water. Whole blood was collected via orbital bleeding, allowed to clot at room temperature for 30 min, and centrifuged at 3000 rpm (4 °C, 15 min) to obtain serum. Serum samples and standards were added to a 96-well plate pre-coated with capture antibody. Following sequential steps of incubation, washing, biotinylated detection antibody addition, enzyme-labeled streptavidin incubation, chromogenic substrate reaction, and stop solution, absorbance was measured at 450 nm using a microplate reader. Target molecule concentrations were calculated based on the standard curve.

### 2.23. HE Staining, Nissl Staining, and Neun Staining

Brain tissue was fixed in 4% paraformaldehyde (PFA) at 4 °C for 24 h, followed by dehydration, embedding, and sectioning into slices of 4–5 μm thickness. After dewaxing and rehydration, sections were stained with hematoxylin-eosin (H&E) for 8–10 min for general histopathological evaluation, and with toluidine blue solution at 60 °C for 20 min to assess Nissl bodies and neuronal morphology. Hippocampal pathological and morphological changes were observed under a light microscope [41,42].

For immunofluorescence, sections were permeabilized with 0.3% Triton X-100 (room temperature, 30 min), blocked with 5% BSA containing 10% normal goat serum (37 °C, 1 h), and incubated with primary antibody (4 °C, overnight) followed by secondary antibody (37 °C, 2 h). Nuclei were counterstained with DAPI. Confocal imaging was performed on a Nikon ECLIPSE Ti2-E microscope (Minato-ku, Tokyo, Japan) using 405, 488, 561, and 640 nm laser lines and a 60× oil-immersion objective. Sequential scanning prevented channel crosstalk. Z-stacks were acquired at 0.5 μm intervals, and maximum intensity projections were generated with NIS-Elements software (v5.21). Imaging parameters were held constant across groups [43].

### 2.24. Immunohistochemical

Hippocampal tissues were fixed in 4% paraformaldehyde, dehydrated, embedded, and cut into 4–5 μm paraffin sections. After deparaffinization, rehydration, and antigen retrieval, sections were blocked with 5% BSA and 10% normal goat serum (37 °C, 30 min), then incubated with primary antibody (4 °C, overnight) followed by secondary antibody (room temperature, 30 min). Immunoreactivity was visualized using DAB chromogen, and images were captured under an optical microscope [44].

### 2.25. Western Blot Analysis

Hippocampal tissues were lysed for protein extraction. Protein concentrations were determined using a BCA assay, and equal amounts were separated by SDS-PAGE, then transferred onto PVDF membranes. Membranes were blocked with 5% non-fat milk in TBST (room temperature, 1 h) and incubated with primary antibody (4 °C, overnight). After washing, membranes were incubated with HRP-conjugated secondary antibody (room temperature, 1 h). Protein bands were visualized using an enhanced chemiluminescence (ECL) reagent kit and a chemiluminescence imaging system (Analytik Jena, Jena, Germany). Band intensities were quantified using ImageJ (version 1.54f, National Institutes of Health, Bethesda, MD, USA) [45].

### 2.26. Immunofluorescence

Hippocampal sections were permeabilized with 0.5% Triton X-100 in PBS (room temperature, 30 min), washed three times with PBS, and blocked with 5% BSA in PBS (room temperature, 1 h). Sections were then incubated with primary antibody in blocking buffer (4 °C, overnight), washed, and incubated with fluorophore-conjugated secondary antibody (room temperature, 1 h, dark). After washing, nuclei were counterstained with DAPI (1 µg/mL, 15 min). Sections were mounted with anti-fade medium and imaged using a Nikon A1R HD confocal microscope with a 60× oil immersion objective. Laser lines (405 nm, 488 nm, 561 nm) were selected accordingly. Images were acquired with NIS-Elements AR software v6.10 and processed using ImageJ (version 1.54f, National Institutes of Health, USA) [46].

### 2.27. Transmission Electron Microscopy

Hippocampal tissue was perfused and fixed with 2.5% glutaraldehyde and 2% paraformaldehyde, then post-fixed in 2.5% glutaraldehyde at 4 °C for 24 h. After dehydration and staining, samples were embedded, ultrathin-sectioned (1 μm), and stained with uranyl acetate (30 min, protected from light) followed by lead citrate (15 min). Mitochondrial morphology was examined using a transmission electron microscope (TEM) (HITACHI HT7800, Velox software v2.11, Japan) [47].

### 2.28. Reverse Transcription (RT) and Real-Time Quantitative PCR

Hippocampal tissues were snap-frozen in liquid nitrogen and stored at −80 °C. Total RNA was extracted using TRIzol reagent per the manufacturer’s protocol. RNA integrity was verified using an Agilent Bioanalyzer 2100 with Agilent RNA 6000 Nano Kit, analyzed via Agilent 2100 Bioanalyzer Expert Software (v2.9, Agilent Technologies, Santa Clara, CA, USA). Reverse transcription was performed with the PrimeScript™ RT Master Mix (Takara, RR036A, Kusatsu, Japan). qPCR was conducted on a StepOnePlus system using SYBR Green Master Mix. Thermal cycling conditions were: 95 °C for 30 s, followed by 40 cycles of 95 °C for 5 s and 60 °C for 30 s. Melting curve analysis confirmed specificity. Relative mRNA expression was calculated by the 2^−ΔΔCt^ method, with GAPDH as the internal reference [48]. All primers were designed and validated by Servicebio Biotechnology Co, Ltd., (Wuhan, China) and sequences are provided in Table 2.

### 2.29. Statistical Analysis

All statistical analyses were performed using GraphPad Prism 8 (San Diego, CA, USA). Results are presented as mean ± standard deviation (SD). Group differences were evaluated by one-way analysis of variance (ANOVA). A *p*-value less than 0.05 was considered statistically significant.

## 3. Results

### 3.1. Optimization of Sugarcane Polyphenol Extraction and Purification by Response Surface Methodology

**Figure 1:**
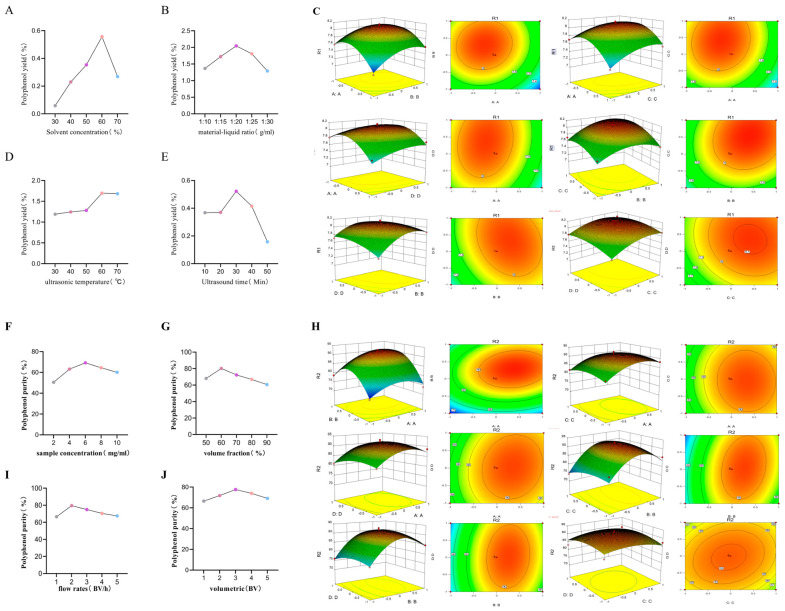
(**A**–**E**) Single-factor experiments and response surface results showing the effects of ethanol concentration, solid-to-liquid ratio, ultrasonic temperature, and time on polyphenol yield. (**F**–**J**) Single-factor experiments and response surface results showing the effects of sample concentration, ethanol volume fraction, and elution conditions on polyphenol purity.

As shown in Figure 1A,B,D,E,I, the highest polyphenol yield was achieved at a solution concentration of 60%, a material-liquid ratio of 1:20, a sonication temperature of 60 °C and a sonication time of 30 min.

For the experimental design, see Appendix A. Response surface regression analysis was carried out on the polyphenol yield of sugarcane peel to obtain the fitted equation:

Y = 8.08 + 0.15 × B + 0.13 × C + 0.085 × D + 0.044 × AB + 0.043 × AC + 0.048 × AD + 0.056 × BC − 0.069 × BD − 0.030 × CD − 0.30 × A^2^ − 0.23 × B^2^ − 0.15 × C^2^ − 0.1 × D^2^

From the above regression model, the optimal process conditions were found to be 62.275% ethanol concentration, 1:17.229 material-liquid ratio, 61.791 °C extraction temperature and 37.892 min sonication time, and the predicted value of sugarcane polyphenol yield was 7.920%. Considering the practical practicability, the extraction conditions were adjusted as follows: ethanol concentration of 60%, material-liquid ratio of 1:15, ultrasonication temperature of 60 °C and ultrasonication time of 40 min. The average sugarcane polyphenol yield was 7.866% after repeating the extraction three times with the adjusted conditions, which indicated that the constructed model was correctly fit to the actual situation, and it also proved that the response surface methodology was applicable to the polyphenol extracting process of sugarcane peels for regression analysis and parameter optimization.

As shown in Figure 1C,F–H,J, the highest polyphenol purity was achieved at the upper sample concentration of 6 mg/mL, solution concentration of 60%, eluent flow rate of 2 BV/h and eluent dosage.

Based on the one-factor test, a four-factor, three-level response surface analysis test was designed according to the principles of central combination experimental design. The experimental design is shown in Appendix B. Response surface regression analysis was performed on sugarcane peel purification to obtain the fitted equation:

Y = 91.31 + 2.81 × A + 4.14 × B − 0.16 × C − 0.087 × D + 1.57 × AB − 0.21 × AC + 0.70 × AD + 1.59 × BC + 0.79 × BD + 0.79 × CD − 4.31 × A2 − 8.35 × B2 − 2.21 × C2 − 2.13 × D2

From the above regression model, the optimal process conditions were found to be 8 mg/mL of up-sampling concentration, 60% of ethanol volume fraction, 3 BV/h of eluent flow rate and 3 BV of elution volume, and the predicted value of the purity of sugarcane polyphenols was 87.238%. After three independent extraction replicates, the average polyphenol yield from sugarcane peel was 86.994%. This result indicates that the established model fits the actual extraction process well, demonstrating that response surface methodology is suitable for the regression analysis and parameter optimization of polyphenol purification from sugarcane peels.

### 3.2. Material Identification

**Figure 2:**
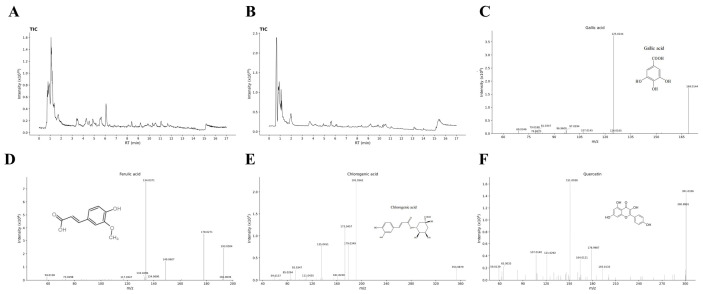
Identification and Analysis of Sugarcane Polyphenols. (**A**) Total ion chromatogram of sugarcane polyphenols (negative spectrum). (**B**) Total ion chromatogram of sugarcane polyphenols (positive spectrum). (**C**) Fragment ion diagram of gallic acid. (**D**) Fragment ion diagram of ferulic acid. (**E**) Fragment ion diagram of chlorogenic acid. (**F**) Fragment ions of quercetin.

Based on liquid chromatography-tandem mass spectrometry (LC-MS/MS) analysis, sugarcane polyphenol extract was found to predominantly contain ten phenolic monomers. By comparing retention times, accurate molecular weights, and characteristic fragment ions from tandem mass spectrometry (MS/MS), these compounds were identified as gallic acid, ferulic acid, chlorogenic acid, rosmarinic acid, protocatechuic acid, quercetin, apigenin, naringin, kaempferol, and isorhamnetin. The structural assignments of these compounds were further confirmed using characteristic fragmentation information provided by MS/MS, establishing a reliable chemical basis for subsequent quantitative analysis and bioactivity studies of the extract. The specific data are annotated in Figure 2 and Table 3.

### 3.3. Effect of SP on Cell Proliferation and Apoptosis

**Figure 3:**
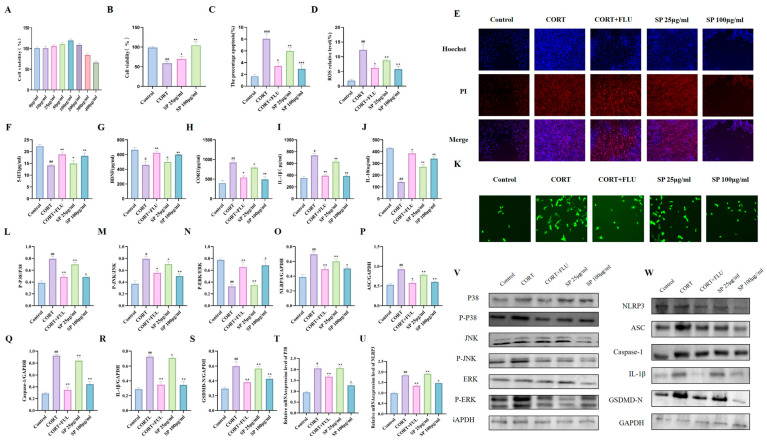
Effect of SP on the viability and apoptosis of neuronal HT22 cells. (**A**) Screening of SP effective dose. (**B**) cell viability assay. (**C**,**E**) Hoechst 33342/PI double staining assay for apoptosis (20×). (**D**,**K**) ROS levels in HT22 cells (20×). (**F**–**J**) Intracellular levels of IL-10, IL-1β, 5-HT, BDNF and CORT. (**L**–**S,V**,**W**) The expression levels of P38, P-P38, JNK, P-JNK, ERK, P-ERK, NLRP3, ASC, Caspase-1, IL-1β and GSDMD-N were detected and quantified. (**T**,**U**) mRNA expression levels of P38 and NLRP3 in cells. All data are expressed as mean ± S.D. # *p* < 0.05, ## *p* < 0.01, ### *p* < 0.001 indicate a statistically significant difference compared to the control group; * *p* < 0.05, ** *p* < 0.01, *** *p* < 0.01 indicate a statistically significant difference compared to the model group.

As shown in Figure 3A,B, to evaluate the neuroprotective effects of sugarcane polyphenols (SP), we examined the impact of different SP concentrations on CORT-induced damage in HT22 cells. Under normal culture conditions, 100 μg/mL SP approached the safe concentration threshold in vitro. However, in the CORT-induced stress model, this concentration exhibited the highest protective benefit, showing the most significant improvement in the survival of damaged cells. Therefore, 100 μg/mL was defined as the high dose for subsequent mechanistic studies to assess its maximum therapeutic potential under pathological conditions.

As shown in Figure 3C,E, compared with the control group, the CORT group exhibited more pronounced apoptotic morphology, such as nuclear condensation and fragmentation. After administration of SP, the apoptotic situation was reversed, and as the dose of SP increased, the apoptotic situation gradually decreased, with the nuclear morphology approaching that of the control group. This indicates that SP (100 μg/mL) can significantly alleviate CORT-induced neuronal death.

As shown in Figure 3F–J, compared with the control group, the levels of 5-HT, BDNF, and IL-10 were significantly reduced in the CORT group, while the levels of CORT and IL-1 were significantly increased. After SP administration, compared with the CORT group, the levels of 5-HT, BDNF, and IL-10 were increased, while the levels of CORT and IL-1 were decreased. In summary, SP can dose-dependently alleviate CORT-induced neuronal death.

### 3.4. Effect of SP on Oxidative Stress

**Figure 4:**
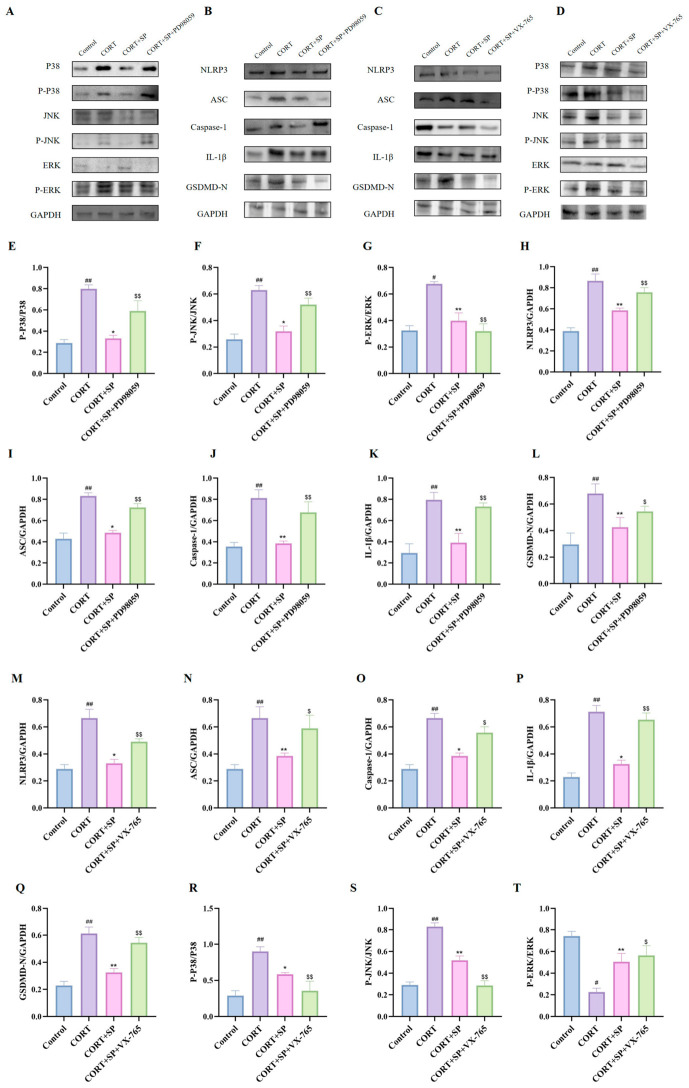
Effect of PD98059 and VX-765 on HT22 cells. (**A**–**T**) The expression levels of P38, P-P38, JNK, P-JNK, ERK, P-ERK, NLRP3, ASC, Caspase-1, IL-1β and GSDMD-N were detected and quantified. All data are expressed as mean ± S.D. # *p* < 0.05, ## *p* < 0.01, indicate a statistically significant difference compared to the control group; * *p* < 0.05, ** *p* < 0.01,indicate a statistically significant difference compared to the model group, $ *p* < 0.05, $$ *p* < 0.01,indicate a statistically significant difference compared to the treatment group.

ROS were labeled with DCFH-DA and observed under a fluorescence microscope. As shown in Figure 4D,K, after incubation with CORT (100 μM) for 24 h, ROS levels significantly increased, while SP treatment resulted in a marked reduction in ROS accumulation. This confirms the ROS-scavenging effect of SP.

### 3.5. The Effect of SP on the Expression of Proteins Related to MAPK and Pyroptosis Signaling Pathways

As shown in Figure 3L-S, compared with the model group, the proportion of P38 protein phosphorylation decreased in cells after SP administration. Compared with the model group, the proportion of JNK protein phosphorylation decreased in cells after SP administration. Compared with the model group, the proportion of ERK protein phosphorylation increased in cells after SP administration. Simultaneously, the mRNA expression level of P38 was reduced.

CORT-induced expression levels of NLRP3, ASC, caspase-1, IL-1β, and GSDMD-N in HT22 neuronal cells were significantly activated. However, SP administration reduced the expression levels of these related proteins and simultaneously decreased NLRP3 mRNA expression levels. This indicates that SP (100 μg/mL) inhibited NLRP3 inflammasome activation and pyroptosis assembly.

### 3.6. Verification of the Effects of SP on MAPK and Pyroptosis Signaling Pathways Using MAPK Inhibitor PD98059 and Pyroptosis Inhibitor VX-765

As shown in Figure 4A,B, compared with the model group, SP (100 μg/mL) reduced the protein expression levels of P38, P-P38, JNK, and P-JNK in HT22 cells. After adding the inhibitor PD98059, the protein expression levels increased significantly. In comparison with the model group, SP (100 μg/mL) increased the protein expression levels of ERK and P-ERK in HT22 cells. After adding the inhibitor PD98059, the protein expression levels were significantly reduced. As predicted, the PD98059 inhibitor validated that SP acts on the MAPK inflammatory pathway in CORT-induced HT22 cells.

Under MAPK inhibitor conditions, the expression levels of NLRP3, ASC, Caspase-1, GSDMD-N, and IL-1β proteins were increased in HT22 cells, and the protective effect of SP was completely abolished. In summary, SP exerts its effects through the MAPK-ERK pathway, blocking the protective effect of ERK and thereby enhancing pyroptosis.

As shown in Figure 4C,D, as compared to the model group, SP (100 μg/mL) reduced the expression levels of NLRP3, ASC, Caspase-1, GSDMD-N, and IL-1β proteins in HT22 cells. Following the addition of the inhibitor VX-765, the protein expression levels significantly increased. As predicted, the VX-765 inhibitor validated that SP acts on the NLRP3 pyroptosis pathway in CORT-induced HT22 cells.

Under pyroptosis inhibitor conditions, the expression levels of ERK and P-ERK proteins increased. The expression levels of P38, P-P38, JNK, and P-JNK proteins decreased. The above results indicate that pyroptosis and the MAPK pathway have a synergistic effect, and blocking pyroptosis can relieve the inhibition of ERK by inflammation.

### 3.7. Results of Behavioral Tests

**Figure 5:**
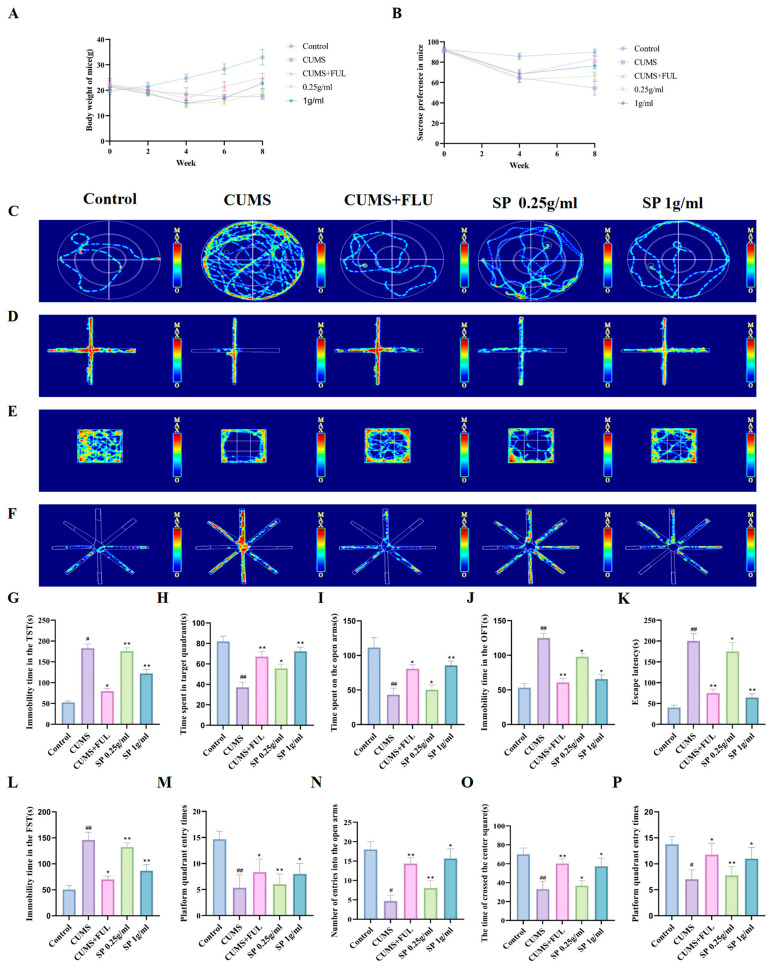
(**A**) Effect of SP on body weight of mice. (**B**) Effect of SP on sucrose preference in mice. (**C**) Thermogram of mouse activity in MWM. (**D**) Thermogram of mouse activity in EPM. (**E**) Mouse activity thermogram in OFT. (**F**) Thermogram of mouse activity in RAM. (**G**,**L**) Mouse immobilization time in TST and FST. (**H**,**M**) Dwell time and traversal times of mice in the MWM target quadrant. (**I**,**N**) Dwell time and number of open-arm stays in the EPM in mice. (**J**,**O**) Dwell time and number of times mice traversed the center in OFT. (**K**,**P**) Activity time and number of crossings of mice in the RAM target quadrant. All data are expressed as mean ± S.D. # *p* < 0.05, ## *p* < 0.01, indicate a statistically significant difference compared to the control group; * *p* < 0.05, ** *p* < 0.01, indicate a statistically significant difference compared to the model group.

Throughout the experiment, the body weight of the control group mice increased steadily. In comparison with the control group, the body weight of the CUMS model group rats decreased significantly. As shown in Figure 5A, the situation improved significantly after SP treatment. Next, the antidepressant effect of SP was evaluated through four behavioral performances.

As shown in Figure 5B, compared with the control group, the sucrose preference score of CUMS model mice was significantly reduced. The MWM test showed that model mice spent less time in the target quadrant than the control group, indicating a decline in cognitive ability. These behavioral alterations were ameliorated following SP treatment. In the elevated plus maze (EPM) test, model mice spent significantly less time in the open arms compared to the control group, suggesting anhedonia and reduced exploratory drive. In the open field test (OFT), model mice exhibited prolonged immobility in the central area, indicating diminished exploratory behavior and core depressive-like symptoms. RAM testing showed that model mice spent more time in the eight-arm maze, indicating severe impairment of learning and memory. However, after SP treatment, learning and memory were restored, exploration time was reduced, the number of crossings decreased, and the mice were able to quickly locate the target position. These findings suggest that SP improves depression-like behavior in mice induced by CUMS.

### 3.8. Levels of IL-10, IL-1β, 5-HT, BDNF, and CORT in Mouse Serum

**Figure 6:**
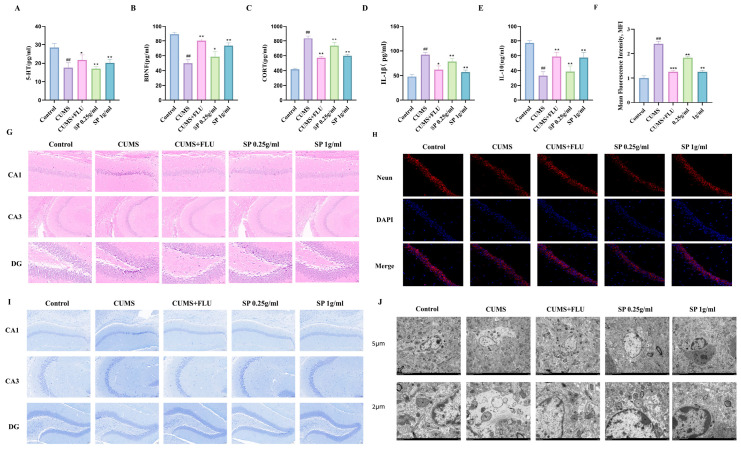
Detection and Staining of Depression-Related Markers. (**A**–**E**) Determination of IL-10, IL-1β, 5-HT, BDNF and CORT in the hippocampus. (**F**–**H**) Neun immunofluorescence image of mouse hippocampal CA1 region (25×) (Red Neun, Blue DAPI). (**G**) HE staining images of hippocampus. (**I**) Images of hippocampal Nissen staining results. (**J**) Transmission electron microscopy results. All data are expressed as mean ± S.D. ## *p* < 0.01,indicate a statistically significant difference compared to the control group; * *p* < 0.05, ** *p* < 0.01, *** *p* < 0.01 indicate a statistically significant difference compared to the model group.

As shown in Figure 6A–E, in comparison with the control group, the levels of 5-HT, BDNF, and IL-10 in the serum of CUMS group mice were significantly reduced. After SP administration, their levels were significantly increased. Compared with the control group, the levels of IL-1β and CORT in the serum of CUMS group mice were significantly increased. After SP treatment, their levels were significantly reduced. These results indicate that SP exerts its antidepressant effects by inhibiting the secretion of inflammatory factors and CORT and increasing the expression of 5-HT and BDNF.

### 3.9. Effects of SP on CUMS-Induced Hippocampal Neuronal Function in Mice

HE staining, as shown in Figure 6G, revealed that compared with the control group, mice in the CUMS group exhibited varying degrees of damage in the CA1, CA3, and DG regions of the hippocampus, manifested as unclear nuclear margins, nuclear shrinkage, pigment aggregation, and disordered cell arrangement. However, this damage was reversed after SP treatment.

Nissl staining, as shown in Figure 6I, as compared with the control group, neurons in the CA1, CA3, and DG regions of the hippocampus in the CUMS group were sparsely arranged with abnormal cell morphology. Neuronal density was reduced, most neurons had decreased volume, and the number of Nissl bodies was reduced. After SP administration, the pathological morphology of neurons in the CA1, CA3, and DG regions of the hippocampus in mice was significantly improved, with regular neuronal morphology. The neurons were arranged in a neat and compact manner, with Nissl bodies clearly visible. The results indicate that neuronal damage improved following SP drug intervention.

As shown in Figure 6H, as compared with the control group, the CUMS group exhibited sparse neuronal arrangement and abnormal cell morphology in the hippocampal CA3 region. Neuronal density was reduced, and most neurons showed decreased volume. After SP administration, the morphological characteristics of neurons in the hippocampal CA3 region of mice improved significantly, with regular neuronal morphology and compact, orderly arrangement. The results indicate that neuronal damage was significantly improved after SP drug intervention.

Transmission electron microscopy revealed the ultrastructure of hippocampal mitochondria, as shown in Figure 6J. In comparison with the control group, the CUMS group exhibited irregular cell morphology in the hippocampus, extensive cytoplasmic edema, and moderate organelle numbers. The cell nucleus was approximately conical in shape, with minimal condensation of heterochromatin and a clear nuclear membrane. Mitochondria were markedly swollen, with intact membrane structures, sparse and dissolved matrix, and broken cristae displaced to the periphery. After SP administration, compared with the model group, the degree of mitochondrial swelling was significantly reduced, with intact membranes, dense matrix and cristae, uniform size, relatively intact membranes, shallow matrix, and reduced incidence of cristae rupture and shortening. These results indicate that SP has a protective effect on CUMS-induced mitochondrial morphological damage in the mouse hippocampus.

### 3.10. Effect of SP on the Expression of MAPK Signaling Pathway-Related Proteins

**Figure 7:**
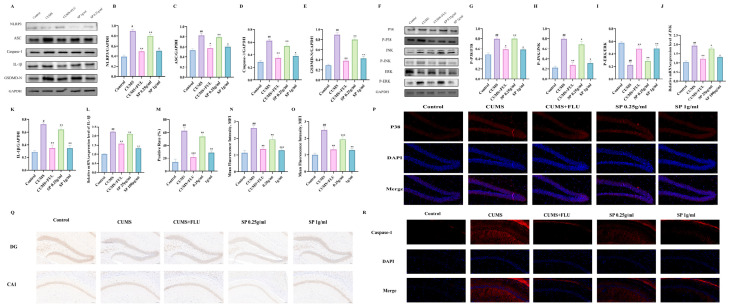
Effects on mitochondrial morphology and expression of MAPK signaling pathway-related proteins in mouse hippocampus. (**A**–**E**,**K**) Detection and quantification of NLRP3, ASC, Caspase-1, IL-1β, and GSDMD-N expression levels. (**F**–**I**) Detection and quantification of P38, P-P38, JNK, P-JNK, ERK, and P-ERK expression levels. (**J**) JNK mRNA expression levels in the hippocampus. (**L**) mRNA expression levels of IL-1β in the hippocampus. (**M**–**Q**) Results of NLRP3 immunohistochemistry (20×). (**O**,**P**) Immunofluorescence intensity of P38 in the DG region (Red P38, Blue DAPI). (**N**–**R**) Immunofluorescence intensity of Caspase-1 in the CA1 region of the hippocampus (20×) (Red Caspase-1, Blue DAPI). All data are expressed as mean ± S.D. # *p* < 0.05, ## *p* < 0.01, indicate a statistically significant difference compared to the control group; * *p* < 0.05, ** *p* < 0.01, *** *p* < 0.001 indicate a statistically significant difference compared to the model group.

As shown in Figure 7F-I, compared with the model group, the proportion of P38 protein phosphorylation in the hippocampus of mice after SP administration was significantly decreased. Compared with the model group, the proportion of JNK protein phosphorylation in the hippocampus of mice after SP administration was decreased. Compared with the model group, the proportion of ERK protein phosphorylation in the hippocampus of mice after SP administration was increased, and the therapeutic effect of SP was significant.

As shown in Figure 7O,P, CUMS induced an increase in the relative fluorescence intensity of P38 in the hippocampus of normal mice. SP treatment reduced the relative fluorescence intensity of P38 and also decreased the mRNA expression level of JNK.

### 3.11. Effects of SP on NLRP3 Inflammatory Vesicle Activation and Pyroptosis

As shown in Figure 7A–K, compared with the CUMS group, SP administration inhibited the activation of the inflammasome and the secretion of NLRP3, ASC, Caspase-1, IL-1β, and GSDMD-N. Meanwhile, SP administration significantly reduced the mRNA expression level of IL-1β. As shown in Figure 7Q,R, the positive signal intensity of NLRP3 and the fluorescence signal intensity of caspase-1 were significantly inhibited after SP administration, with the inhibitory effect of SP being dose-dependent. This indicates that SP inhibited the activation of the NLRP3 inflammasome and pyroptosis assembly in CUMS mice.

### 3.12. Validation of the Effect of SP on the MAPK Inflammatory Pathway Using the MAPK Signaling Pathway Inhibitor PD98059

**Figure 8:**
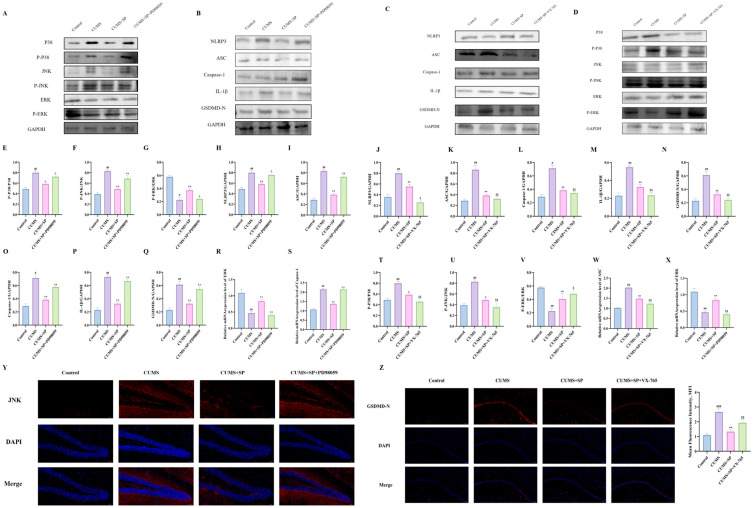
(**A**,**B**,**E**–**I**,**O**–**Q**) Detection and quantification of the expression levels of P38, P-P38, JNK, P-JNK, ERK, P-ERK, NLRP3, ASC, Caspase-1, IL-1β, and GSDMD-N. (**R**,**S**) mRNA expression levels of ERK and Caspase-1 in the hippocampus. (**X**,**Y**) Fluorescence intensity of JNK in the DG region of the hippocampus. (**C**,**D**,**J**–**N**,**T**–**V**) Detection and quantification of NLRP3, ASC, Caspase-1, IL-1β, GSDMD-N, P38, P-P38, JNK, P-JNK, ERK, and P-ERK expression levels. (**W**,**X**) mRNA expression levels of ASC and ERK in the hippocampus. (**Z**) Fluorescence intensity of GSDMD-N in the CA1 region of the hippocampus. All data are expressed as mean ± S.D. # *p* < 0.05, ## *p* < 0.01, ### *p* < 0.001 indicate a statistically significant difference compared to the control group; * *p* < 0.05,** *p* < 0.01, indicate a statistically significant difference compared to the model group, $ *p* < 0.05, $$ *p* < 0.01, indicate a statistically significant difference compared to the treatment group.

As shown in Figure 8A,E–G, compared with the model group, SP treatment reduced the expression levels of P38, P-P38, JNK, and P-JNK proteins in the mouse hippocampus. After adding the inhibitor PD98059, the protein expression levels were significantly increased. Compared with the model group, SP treatment increased the protein expression levels of ERK and P-ERK in the mouse hippocampus. After adding the inhibitor PD98059, the protein expression levels were significantly decreased. Meanwhile, the mRNA expression levels of ERK were significantly increased.

As shown in Figure 8Y, the fluorescence signal intensity of JNK was significantly inhibited after SP administration. After adding the inhibitor PD98059, the fluorescence intensity was significantly enhanced. As predicted, the PD98059 inhibitor validated that SP acts on the MAPK inflammatory pathway in CUMS-induced mice.

As shown in Figure 8B,H,I,O–Q, under MAPK inhibitor conditions, the expression levels of NLRP3, ASC, Caspase-1, GSDMD-N, and IL-1β proteins in the mouse hippocampus were increased, and the protective effect of SP was completely abolished. As shown in Figure 8L, SP treatment significantly reduced the mRNA expression level of Caspase-1. The above results indicate that SP exerts its effects via the MAPK-ERK pathway, and blocking ERK eliminates the anti-pyroptotic effect.

### 3.13. Verification of the Effect of SP on Pyroptosis Using Caspase-1 Inhibitor VX-765

As shown in Figure 8C,J–N, the results indicate that CUMS induces the activation of caspase-1, IL-1β, and GSDMD-N in the hippocampus. SP treatment significantly inhibited the activation of caspase-1, IL-1β, GSDMD-N, and pyroptosis. Following the addition of the inhibitor VX-765, the protein expression levels were significantly increased. Concurrently, the mRNA expression levels of ASC and GSDMD-N were reduced.

As shown in Figure 8Z, SP reduces the fluorescence intensity of GSDMD-N after treatment, and the fluorescence intensity increases after the addition of the inhibitor VX-765. As suspected, VX-765 administration has a synergistic effect on the action of SP in mice.

As shown in Figure 8D,N–U, under pyroptosis inhibitor conditions, the expression levels of ERK and P-ERK proteins increased, while the expression levels of P38, P-P38, JNK, and P-JNK proteins decreased. SP may inhibit NLRP3 inflammasome-mediated pyroptosis by activating the MAPK/ERK pathway; simultaneously, the suppression of pyroptosis and the attenuation of the neuroinflammatory microenvironment may help maintain the sustained activation of ERK signaling, thereby forming a potential self-reinforcing protective loop.

## 4. Discussion

This study provides novel evidence that sugarcane polyphenols (SP) alleviate depressive-like behaviors in CUMS-exposed mice and protect against CORT-induced neuronal injury. Our data delineate a coherent mechanistic model wherein SP’s benefits are mediated through a dual regulation of the MAPK pathway and the NLRP3 inflammasome-pyroptosis axis. Specifically, SP treatment suppressed the activation of the pro-inflammatory p38 and JNK kinases, activated the pro-survival ERK pathway, and concurrently inhibited the assembly and activity of the NLRP3 inflammasome, leading to reduced caspase-1 activation, IL-1β maturation, and GSDMD-mediated pyroptosis.

The observed depressive-like behaviors in the CUMS model, such as reduced body weight and decreased sucrose preference, are consistent with previous reports, validating the efficacy of our modeling approach [49]. Fluoxetine, a widely used classical antidepressant, was selected as the positive control in this study [50]. Our results show that SP intervention significantly increased the sucrose preference rate in the sucrose preference test (SPT), decreased immobility time in the forced swim and tail suspension tests, improved memory function in the Morris water maze (MWM) test, and increased exploratory activity and time spent in the central zone of the elevated plus maze (EPM). This comprehensive amelioration of behavioral indices indicates that SP, similar to fluoxetine, effectively alleviates depressive-like symptoms.

Neuroinflammation plays a pivotal role in the pathogenesis of psychiatric disorders, including depression [51]. Excessive stress and chronic inflammation can lead to neuroinflammation and cellular damage, exacerbating neuropsychiatric disease progression [52]. Our results indicate that CUMS stress induced aberrant expression of 5-HT and BDNF in the mouse hippocampus, promoted the secretion of inflammatory cytokines IL-1β and CORT, reduced the release of the anti-inflammatory cytokine IL-10, and accelerated hippocampal neuronal damage. SP treatment produced effects analogous to those of fluoxetine, namely inhibiting inflammatory cytokine release, restoring neurotrophic factor expression, and repairing hippocampal neuronal damage.

At the cellular level, repeated CORT administration has been reported to cause hypothalamic–pituitary–adrenal (HPA) axis dysfunction, neuronal injury, and the induction of depressive-like behaviors [53]. Therefore, we employed CORT to induce injury in HT22 cells, modeling the pathological process of depression in vitro. Our results confirmed that CORT significantly reduced cell viability, induced apoptosis, and promoted the secretion of inflammatory factors. In contrast, SP treatment attenuated CORT-induced HT22 cell injury to a considerable extent, promoted cell proliferation, and inhibited apoptosis and IL-1β secretion. Furthermore, we observed that CORT impaired mitochondrial function, as evidenced by marked mitochondrial swelling and disruption of membrane structure, whereas SP (100 μg/mL) repaired this structural damage.

Accumulating evidence implicates three members of the MAPK family—p38, JNK, and ERK—in the regulation of depression [54,55]. Consistent with our observations, several reports have shown a significant increase in the phosphorylation levels of JNK and p38 alongside a significant decrease in ERK phosphorylation in the hippocampus of rodent models following CUMS induction [56]. Studies report that various polyphenols can modulate the MAPK signaling pathway by acting at different steps in its activation process [57]. For instance, kaempferol, lignans, salicin, and apigenin can inhibit the activity of JNK, p38, and ERK, thereby suppressing TNF-α-stimulated ICAM1 expression in respiratory epithelial cells [58,59,60]. Similarly, our study found that SP treatment blocked the CUMS-induced increase in phosphorylated p38/JNK levels while significantly elevating phospho-ERK1/2 levels. This suggests that the antidepressant effect of SP may largely depend on the inhibition of the p38/JNK signaling pathways. Notably, the ERK pathway is a key branch of the MAPK cascade. The ERK inhibitor PD98059 reversed the anti-pyroptotic and neuroprotective effects of SP, demonstrating that ERK1/2 activation plays a critical role and is necessary for SP’s efficacy in treating neuroinflammation-induced depression.

NLRP3 is an inflammasome sensor protein that has been extensively studied in the context of various diseases [61]. Activation of NLRP3 leads to the formation of an oligomeric complex, including ASC and caspase-1, termed the “NLRP3 inflammasome.” Its activation is a key regulator of cellular pyroptosis, promoting caspase-1 activation, which subsequently cleaves GSDMD into its pore-forming N-terminal fragment (GSDMD-N). This ultimately leads to cell lysis and the release of LDH and pro-inflammatory cytokines, representing a form of caspase-1-dependent programmed cell death (pyroptosis) [62,63].

SP treatment consistently inhibited NLRP3 inflammasome activation and downregulated NLRP3, ASC, pro-caspase-1, caspase-1, IL-1β, and GSDMD-N expression in both CUMS mouse hippocampus and CORT-stimulated HT22 cells, thereby suppressing pyroptosis and inflammatory factor release. A synergistic effect was observed between SP and the pyroptosis inhibitor VX-765. NLRP3 inflammasome activation is associated with ROS accumulation, and conversely, its activation can induce intracellular ROS production. Our results showed that SP scavenged ROS in HT22 cells, suggesting that SP may inhibit NLRP3 inflammasome activation by reducing ROS accumulation. Furthermore, VX-765 treatment, while inhibiting pyroptosis, also influenced the expression levels of MAPK-related proteins, exhibiting a synergistic effect with SP. This supports the existence of crosstalk between the MAPK signaling and NLRP3 inflammasome/pyroptosis pathways.

In conclusion, our study suggests that the MAPK/NLRP3 axis may be a potential therapeutic target for depression. SP can ameliorate depressive symptoms by modulating the interaction between MAPK signaling and NLRP3-mediated pyroptosis. Mechanistically, SP-activated ERK signaling is crucial for inhibiting the NLRP3 inflammasome and pyroptosis, while the inhibition of pyroptosis, in turn, can relieve the inflammatory suppression of ERK signaling, forming a beneficial feedback loop that alleviates neuroinflammation and depressive-like behaviors. Future research could focus on identifying the specific active monomeric compounds in SP, their blood–brain barrier permeability, and the role of this signaling axis in broader models of depression to facilitate its translation into clinical applications.

## 5. Conclusions

In summary, the results of this study demonstrate that SP exerts its effects on reducing pyroptosis by dual-regulating the MAPK-pyroptosis axis: it inhibits p38/JNK to block the activation of the NLRP3 inflammasome. Meanwhile, SP activates ERK to promote neural repair, effectively alleviating neuroinflammation and oxidative stress damage. These findings provide a theoretical basis and experimental foundation for the development of new antidepressant drugs with fewer side effects. Additionally, the research data obtained will further advance the development of SP’s efficacy and activity, and promote the diversified expansion of SP’s applications.

## Figures and Tables

**Table 1:** Mold Making Schedule.

Weekly	1 Day	2 Day	3 Day	4 Day	5 Day	6 Day	7 Day
Week 1	Tail clamping: 5 min	Food deprivation: 24 h	Noise exposure: 12 h	Water deprivation: 24 h	Damp bedding: 24 h	Warm water swimming (28 °C): 10 min	Cage tilt (45°): 12 h
Week 2	Cage tilt (45°): 12 h	Water deprivation: 24 h	Novel object exposure: 12 h	Cage shaking: 10 min	Reversed light/dark cycle: 12 h	Food deprivation: 24 h	Ice-cold water swimming (4 °C): 5 min
Week 3	Restraint: 1 h	Cage shaking: 10 min	Noise exposure: 12 h	Damp bedding: 24 h	Novel object exposure: 12 h	Food deprivation: 24 h	Tail clamping: 5 min
Week 4	Ice-cold water swimming (4 °C): 5 min	Damp bedding: 24 h	Reversed light/dark cycle: 12 h	Water deprivation: 24 h	Cage shaking: 10 min	Tail clamping: 5 min	Restraint: 1 h
Week 5	Tail clamping: 5 min	Food deprivation: 24 h	Noise exposure: 12 h	Water deprivation: 24 h	Damp bedding: 24 h	Warm water swimming (28 °C): 10 min	Novel object exposure: 12 h
Week 6	Cage tilt (45°): 12 h	Water deprivation: 24 h	Novel object exposure: 12 h	Cage shaking: 10 min	Reversed light/dark cycle: 12 h	Food deprivation: 24 h	Ice-cold water swimming (4 °C): 5 min
Week 7	Restraint: 1 h	Cage shaking: 10 min	Tail clamping: 5 min	Noise exposure: 12 h	Damp bedding: 24 h	Novel object exposure: 12 h	Food deprivation: 24 h
Week 8	Ice-cold water swimming (4 °C): 5 min	Damp bedding: 24 h	Reversed light/dark cycle: 12 h	Water deprivation: 24 h	Cage shaking: 10 min	Tail clamping: 5 min	Restraint: 1 h

**Table 2:** Primer sequence information table.

	Forward	Reverse
GAPDH	CCTCGTCCCGTAGACAAAATG	TGAGGTCAATGAAGGGGTCGT
P38	TGAGCCTGTTGCTGACCCTTAT	CAGGTGCTCAGGACTCCATTTC
ERK	CCAGGAAAGCATTACCTTGACC	CCAGAGCCTGTTCAACTTCAATC
JNK	GCTGGAATTATTCATCGGGACT	AGTCACCACATAAGGCGTCATC
NLRP3	ATGACTTTCCAGGAGTTCTTCGC	CCAAAGAGGAATCGGACAACAA
ASC	CAGCACAGGCAAGCACTCATT	TCATCTTGTCTTGGCTGGTGG
Caspase-1	AAAGACAAGCCCAAGGTGATC	CCAAGTCACAAGACCAGGCATA
Il-1β	GCTTCAGGCAGGCAGTATCA	AATGGGAACGTCACACACCA
GSDMD-N	GACTCTGGAGAACTGGTGCC	TTCCAAGACGTGCTTCACCA

**Table 3:** Mass spectrometry analysis parameters for polyphenolic compounds.

No.	Polyphenolic Compounds	Molecular Formula	Retention Time/min	Actual Value (*m*/*z*)	Theoretical Value (*m*/*z*)	Fragment Ions (*m*/*z*)
1	Gallic acid	C_7_H_6_O_5_	1.76	169.0144	169.0142	125, 169, 81, 97, 79
2	Ferulic acid	C_10_H_11_NO_3_	7.06	176.0706	176.0706	130, 145, 117, 106, 149
3	Chlorogenic acid	C_16_H_18_O_9_	4.05	353.0885	353.0878	191, 135, 179, 173, 99
4	Rosmarinic acid	C_18_H_16_O_8_	6.51	359.0778	359.0772	161, 171, 197, 133, 135
5	Protocatechuic acid	C_7_H_6_O_4_	3.51	153.0195	153.0193	109, 153, 59, 108, 117
6	Quercetin	C_26_H_28_O_15_	6.21	579.1396	579.1355	491, 116, 313, 313, 271
7	Apigenin	C_15_H_10_O_5_	8.13	269.0455	269.0456	269, 117, 89, 96, 59
8	Naringenin	C_21_H_18_O_8_	3.04	503.1199	503.1193	145, 189, 251, 131, 59
9	Kaempferol	C_15_H_10_O_6_	7.55	285.0405	285.0404	285, 133, 241, 96, 159
10	Isorhamnetin	C_16_H_12_O_7_	7.58	315.0511	315.0510	300, 315, 272, 299, 314

## Data Availability

Data will be made available on request.

## Abbreviations

SP, sugarcane polyphenols; CUMS, chronic unpredictable mild stress; PVDF, polyvinylidene fluoride; PFA, polyformaldehyde; BSA, bovine serum albumin; TST, tail suspension test; FST, forced swim test; OFT, open field test; CORT, corticosterone; MWM, Morris Water Maze; IL-6, interleukin-6; IL-1β, interleukin-1β; FLU, fluoxetine; 5-HT, serotonin; BDNF, brain-derived neurotrophic factor; RAM, Radial Arm Maze; HE, hematoxylin and eosin staining; TEM, transmission electron microscopy; NLRP3, NLR family pyrin domain containing 3; ASC, Apoptosis-associated speck-like protein containing a CARD; Caspase-1, cysteinyl aspartate specific proteinase; EPM, elevated plus maze; GSDMD-N, GasderminD-N; MAPK, Mitogen-Activated Protein Kinases; P38, VX-765, belnacasan; P38 Mitogen-Activated Protein Kinases; JNK, c-Jun N-terminal Kinase; FBS, fetal bovine serum; ERK, Extracellular Signal-Regulated Kinase; SPT, sucrose preference test; WB, Western Blot.

## Appendix A

**Table A1:** Response surface test factor level table.

Horizontal Factors	X_1_ (%)	X_2_ (g/mL)	X_3_ (°C)	X_4_ (min)
−1	50	1:10	50	20
0	60	1:20	60	30
1	70	1:30	70	40

X_1_, X_2_, X_3_, and X_4_ are the coded values for solvent concentration, feed-to-solvent ratio, ultrasonication temperature, and ultrasonication time, respectively.

**Table A2:** Response surface design and results.

No.	X_1_	X_2_	X_3_	X_4_	Y_1_
	(%)	(g/mL)	(°C)	(min)	(%)
1	−1	−1	0	0	7.595
2	1	−1	0	0	7.132
3	−1	1	0	0	7.872
4	1	1	0	0	7.584
5	0	0	−1	−1	7.552
6	0	0	1	−1	7.903
7	0	0	−1	1	7.807
8	0	0	1	1	8.04
9	−1	0	0	−1	7.793
10	1	0	0	−1	7.444
11	−1	0	0	1	7.862
12	1	0	0	1	7.703
13	0	−1	−1	0	7.545
14	0	1	−1	0	7.64
15	0	−1	1	0	7.708
16	0	1	1	0	8.028
17	−1	0	−1	0	7.702
18	1	0	−1	0	7.249
19	−1	0	1	0	7.852
20	1	0	1	0	7.57
21	0	−1	0	−1	7.42
22	0	1	0	−1	7.868
23	0	−1	0	1	7.708
24	0	1	0	1	7.88
25	0	0	0	0	8.144
26	0	0	0	0	8.022
27	0	0	0	0	8.172
28	0	0	0	0	7.99
29	0	0	0	0	8.055

## Appendix B

**Table A3:** Design and results of purified response surfaces.

Horizontal Factors	X_5_ (mg/mL)	X_6_ (%)	X_7_ (BV/h)	X_8_ (BV)
−1	4	50	1	2
0	6	60	2	3
1	8	70	3	4

X_5_, X_6_, X_7_ and X_8_ are coded values for up-sampling concentration, eluent concentration, eluent flow rate and eluent dosage, respectively.

**Table A4:** Response surface design and results.

No.	X_5_	X_6_	X_7_	X_8_	Y_2_
	(mg/mL)	(%)	(BV/h)	(BV)	(%)
1	−1	−1	0	0	72.78
2	1	−1	0	0	73.877
3	−1	1	0	0	78.534
4	1	1	0	0	85.909
5	0	0	−1	−1	85.219
6	0	0	1	−1	87.502
7	0	0	−1	1	83.083
8	0	0	1	1	88.539
9	−1	0	0	−1	82.498
10	1	0	0	−1	88.654
11	−1	0	0	1	80.943
12	1	0	0	1	89.91
13	0	−1	−1	0	81.423
14	0	1	−1	0	85.412
15	0	−1	1	0	74.193
16	0	1	1	0	84.534
17	−1	0	−1	0	82.675
18	1	0	−1	0	88.18
19	−1	0	1	0	82.324
20	1	0	1	0	86.987
21	0	−1	0	−1	77.389
22	0	1	0	−1	84.59
23	0	−1	0	1	75.987
24	0	1	0	1	86.342
25	0	0	0	0	89.312
26	0	0	0	0	92.215
27	0	0	0	0	93.123
28	0	0	0	0	91.026
29	0	0	0	0	90.874
